# The Significant Pathways and Genes Underlying the Colon Cancer Treatment by the Traditional Chinese Medicine PHY906

**DOI:** 10.1155/2017/8753815

**Published:** 2017-05-15

**Authors:** Ziyuan Su, Changyu Zhou, Shaoyou Qin, Erna Jia, Zhenting Wu

**Affiliations:** ^1^Research Center of TCM, The Affiliated Hospital of Changchun University of Traditional Chinese Medicine, Changchun, China; ^2^Digest Department, China-Japan Union Hospital of Jilin University, Changchun, China; ^3^Digest Department, Heilongjiang Provincial Hospital, Heilongjiang, China

## Abstract

**Background:**

We attempted to explore the molecular mechanism underlying PHY906 intervention of colon cancer.

**Methods:**

The microarray data of tumors treated by PHY906 and PBS alone were downloaded from the public Gene Expression Omnibus database. The dataset was further analyzed for the differentially expressed genes (DEGs) and their related biological functions were analyzed, followed by function and pathways. Protein-protein interaction (PPI) network was constructed and the significant nodes were screened by network centralities and then the significant modules analysis. Besides, they were clustered and transcriptional factors (TFs) were predicted.

**Results:**

The gene expression patterns changed induced by PHY906 treatment, including 414 upregulated and 337 downregulated DEGs. The biological process of response to steroid hormone stimulus and regulation of interferon-gamma production were significantly enriched by DEGs. Ezh2 (enhancer of zeste 2) was found to be the key node in PPI network. There are 12 significant TFs predicted for module 1 genes and 3 TFs for module 2 genes.

**Conclusions:**

PHY906 treatment may function in protecting the epithelial barrier against tumor cell invasion by modulating IFN-*γ* level and mediating cancer cell death by activating the response to steroid hormone stimulus and activating the response to steroid hormone stimulus. E2f1, Hsfy2, and Nfyb may be therapeutic targets for colon cancer. PHY906 showed treatment efficacy in modulating cell apoptosis by intervening interferon-gamma production and response to steroid hormone stimulus. Ezh2 and its TFs such as E2f1, Hsfy2, and Nfyb may be the potential therapeutic targets for anticancer agents development.

## 1. Background 

Colon cancer is the common tumor derived from gastrointestinal systems, which has affected 1.4 million new cases in 2012 and results in 694,000 deaths globally [1]. It has been proposed to be the third most common cancer of all cases and the second most common cause of cancer related deaths [2]. The standard treatment used for colon cancer is the combination of surgery, chemotherapy, or radiation therapy [3]. The survival rate for cases after treatment is poor, which may be attributed to the treatment-related toxicity. The traditional Chinese medicine (TCM) as the adjuvant treatment plays a critical role in decreasing the complications of chemotherapy and radiation therapy in colon cancer patients [3].

PHY906 is a TCM prepared from four medical herbs, such as* Glycyrrhiza uralensis* Fisch. (G),* Paeonia lactiflora* Pall. (P),* Scutellaria baicalensis* Georgi (S), and Ziziphus jujuba Mill. (Z). It has been widely used for treating gastrointestinal symptoms in Eastern countries for about 1800 years [4]. In recent years, the four-herb TCM has been suggested to be used for cancer therapy for its potent anticancer properties [5]. It plays a modulator role in gastrointestinal toxicity induced by the chemotherapeutic drug irinotecan (CPT-11) of patients with colorectal cancer [6]. However, the mechanism underlying the antitumor modulation of PHY906 in chemotherapy has not been suggested clearly.

Currently, microarray technology has been widely applied to elucidate the tumor progression in different conditions. Wang et al. developed the microarray data of mice bearing colon tumor following treatment with PHY906 plus CPT-11 and suggested that the combination of PHY906 and CPT-11 created a unique response in colon tumors [7].

In order to explore the potential mechanism of PHY906 in treating colon tumors, we reanalyzed the published microarray data of colon tumor treated with PHY906 and PBS (Phosphate Buffered Saline) individually. The differentially expressed genes (DEGs) induced by PHY906 intervention were identified and then the DEGs related function and pathways were analyzed.

## 2. Materials and Methods 

### 2.1. Microarray Data Acquisition

In order to evaluate the effect of PHY906 on colon cancer, we mined the gene expression data from fully public GEO database. The microarray data with the series number GSE25192 were downloaded from GEO database, which was contributed by Wang et al. [7]. The microarray data included 32 colon tumor samples from tumor-bearing mice treated with Phosphate Buffered Saline (PBS) (*n* = 10), PHY-906 (*n* = 9), CPT-1 (*n* = 10), and the combination of PHY-906 and CPT-1 (*n* = 9). In order to explore the therapeutic effect of PHY-906, we only selected the dataset of 9 samples of BDF-1 mice bearing colon 38 tumors treated with PHY906 and 10 tumor samples of those treated with PBS as controls for further analysis. The raw data were downloaded based on the platform of CCDTM Mm-CCDTM36k (derived from GPL5960).

### 2.2. Data Preprocessing and DEG Analysis

The raw file data (in gpr format) were preprocessed in limma package [8], including background correction, expression normalization, and microarray data condensation. Then, the DEGs in PHY906 treated samples compared with PBS samples were analyzed. *P* values for DEGs were calculated with nonpaired *t*-test with the application of limma package. For DEGs identification, the cutoff value was set at *P* < 0.05 and |log_2_⁡FC(fold change)| ≥ 0.58.

### 2.3. Function Enrichment Analysis

The gene sets that share the common biological function and pathways can be analyzed by DAVID online tool [9]. In the present study, the up- and downregulated DEGs were subjected to function enrichment analysis, respectively. The count ≥ 2 and *P* value < 0.05 were set as the cutoff value.

### 2.4. Protein-Protein Interaction Network and Significant Nodes Analysis

The protein-protein interactions (PPIs) were predicted based on the information deposited in STRING (Search Tool for the Retrieval of Interacting Genes/Proteins) database [10]. The protein pairs collection was based on the evidence of neighborhood, gene fusion, cooccurrence, coexpression experiments, databases, and text mining. The gene sets with differential expression were submitted to STRING online tool. The species was set as Mus and PPI score (medium confidence) as 0.4. The PPI network comprised of DEG nodes was visualized by Cytoscape software.

The essential nodes within PPI network were analyzed based on 3 network centralities, such as degree centrality [11], betweenness centrality [12], and subgraph centrality [13]. The multiple centrality measures were conducted by CytoNCA plugin [14]. Degree and subgraph were calculated to evaluate the significance of network nodes. In addition, the betweenness was obtained to measure the influence of a node on the spread of information throughout the network.

### 2.5. Module Analysis

The gene sets in the modules of network generally share the common biological process. The significant modules in PPI network were analyzed by hierarchical cluster analysis with the application of ClusterONE [15] and the KEGG (Kyoto Encyclopedia of Genes and Genomes) pathways for module genes were annotated. The modules with *P* < 2.0*E* − 6 were considered as significant.

### 2.6. Prediction of Transcription Factors for Module Genes

The TFs that played a regulatory role in module were predicted by iRegulon Cytoscape plugin [16]. The TF-target gene interactions were collected from Transfac, Jaspar, Encode, Swissregulon, and Homer database. The significant TFs were analyzed based on the TF binding motif enrichment analysis. The parameters were set as follows: minimum identity between orthologous genes: 0.05 and maximum false discovery rate on motif similarity: 0.001. Finally, the Normalized Enrichment Score (NES) was output. TF-target gene pairs with NES > 5 were selected and visualized in PPI network modules.

## 3. Results 

### 3.1. DEGs Identification

**Figure 1:**
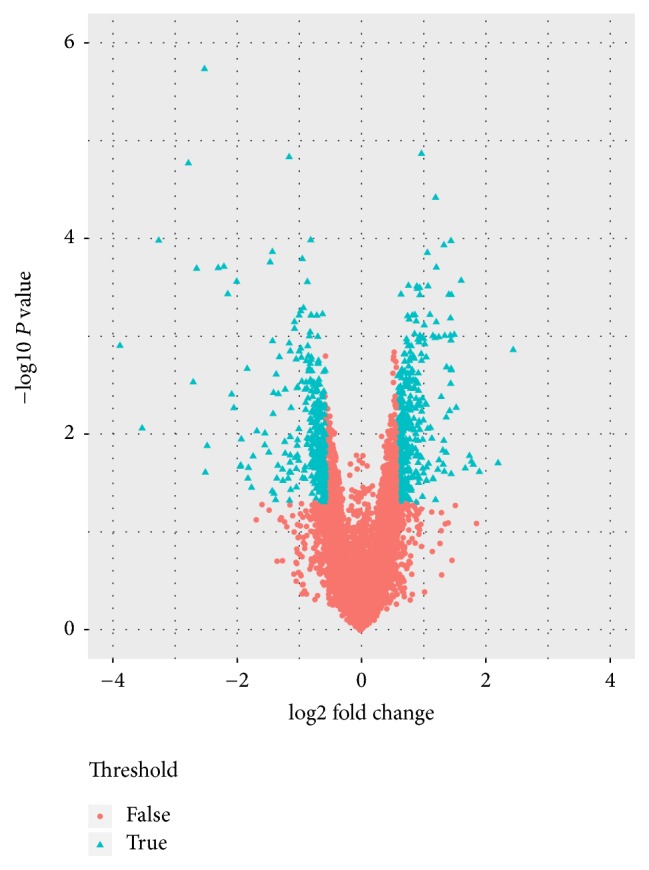
Volcano plot of the differential expression of genes.

Total 751 DEGs were identified in PHY906 treated colon tumor tissues, including 414 up- and 337 downregulated DEGs. As shown in Figure 1, volcano plot illustrates that the expression patterns of genes are altered in tumors after PHY906 treatment compared with PBS treatment.

### 3.2. Function Enrichment Results

The DEGs were enriched in 3 GO Categories, such as biological process (BP), cellar component (CC), and molecular function (MF) (Table 1). The upregulated DEGs were closely related to response to steroid hormone stimulus related BP, proteinaceous extracellular matrix related CC, and di- and trivalent inorganic cation transmembrane transporter activity related MF. The downregulated genes were significantly enriched in regulation of interferon-gamma production (BP), cell fraction (CC), and cation:amino acid symporter activity (MF). The significant pathway for downregulated DEGs was tryptophan metabolism, while there was no pathway enriched by upregulated DEGs.

### 3.3. PPI Network and Significant Nodes

**Figure 2:**
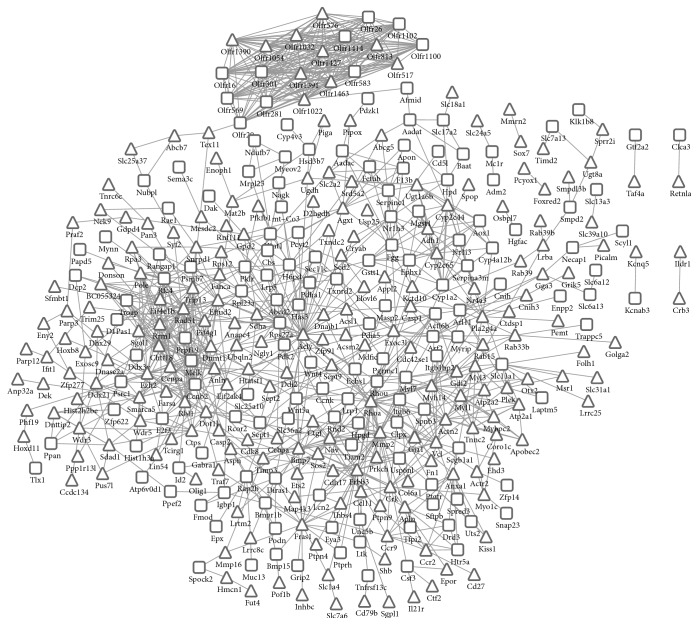
The protein-protein interaction network for differentially expressed genes. The trigon represents upregulated genes and the square represents downregulated genes.

Based on the protein interactions corresponding to DEGs, the PPI network was constructed, which was comprised of 365 DEGs and 1005 protein pairs (Figure 2). Then, the key nodes in PPI network were analyzed by degree, betweenness, and subgraph centrality. As shown in Table 2, Acly (ATP citrate lyase), Rhoa (ras homolog family member A), Erbb3 (erb-b2 receptor tyrosine kinase 3), Ezh2 (enhancer of zeste 2), and Rps27a (ribosomal protein S27a) are the common key nodes according to both degree and betweenness. Olfr20 (olfactory receptor 20) and Olfr1022 (olfactory receptor 1022) are the significant nodes with high scores based on subgraph and degree.

### 3.4. Modules and TFs

After cluster analysis, 2 significant modules were screened. Function enrichment analysis showed that the module 1 genes were closely related to sensory perception of smell, sensory perception of chemical stimulus related biological process, olfactory receptor activity related MF, and olfactory transduction pathway. The module 2 genes were significantly enriched in cell cycle and DNA replication related BP and pathways (Table 3).

**Figure 3:**
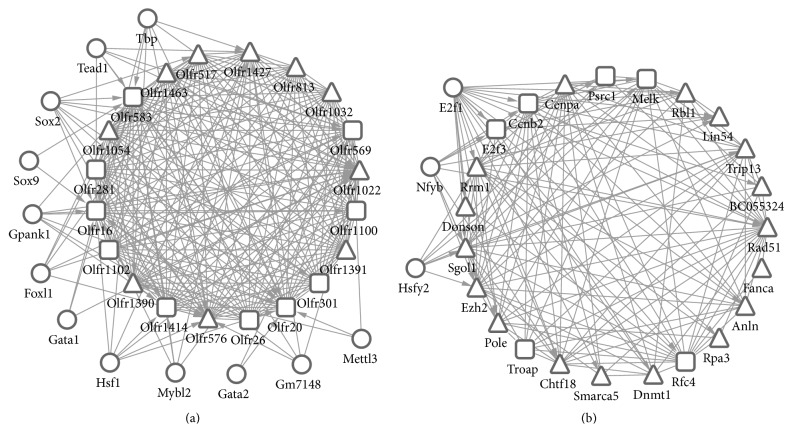
The significant modules and their related TFs. (a) Module 1; (b) module 2; TFs: transcriptional factors. The trigon represents upregulated genes and the square represents downregulated genes. The roundness means the transcriptional factors.

Figure 3 illustrates that there are 12 significant TFs and 62 TF-target gene pairs in module 1. In module 2, 3 TFs are predicted for module genes and involved in 34 TF-target pairs (Figure 3).

## 4. Discussion 

PHY906, as one of the traditional Chinese herbal formulations, possesses the advantage in decreasing the side effect and complications following chemo- and radiotherapies [17]. PHY906 has been commonly used as the adjuvant agent for treatment of various cancers, especially for colon cancer. The understanding of the role of PHY906 in colon cancer treatment is insufficient from current knowledge. In order to provide novel insight to understand the molecular mechanism of PHY906 in treating colon cancer, the microarray data from the colon tumors treated with PHY906 alone were further analyzed.

Our data demonstrated that the PHY906 administration induced significant alterations in gene expression levels of colon tumors comparable to PBS treatment. In the presents study, total 751 DEGs were screened out in PHY906 treated tumors including 414 upregulated DEGs and 337 downregulated ones. Function analysis showed the regulation of interferon-gamma production and response to steroid hormone stimulus were dysregulated in PHY906 treated tumors.

The biological process of regulation of interferon-gamma (IFN-*γ*) production is significantly enriched by downregulated genes. It has been reported that IFN-*γ* affects the barrier function of epithelial monolayers [18]. The concentration dependent experiment has shown that IFN in high concentration diminishes the monolayer resistance by increasing the tight junction permeability [18]. IFN-*γ* has been found to affect the actin distribution and tight junction permeability is affected by cytoskeletal rearrangements [19]. IFN-*γ* may decrease the epithelial barrier function by rearranging cytoskeletal actin. It is in line with our finding that the actin cytoskeleton was activated by upregulated genes in PHY906 treated colon tumors. Our work also found that IFN-*γ* production was decreased by involvement with downregulated genes, which could illustrate that PHY906 treatment protected the epithelial barrier against tumor cell invasion by modulating IFN-*γ* level.

In this paper, response to steroid hormone stimulus was significantly enriched by upregulated genes. Steroid hormone is suggested to be a major regulator in cell apoptosis [20]. Previous data has indicated that antiprogestins initiate terminal differentiation and contribute to cell death in breast cancer cells [21]. A line of evidence also shows that apoptosis plays a critical role in tumor cells response to the therapy of cancer [22]. The apoptotic response is determined by the response of tumor cells to the susceptibility of antitumor therapies such as radiation and chemotherapy [23]. The heterogeneity of antitumor drug susceptibility is well correlated with genetic and hormonal regulation and the apoptotic response is initiated by various hormone stimuli [20]. Our work suggested that steroid hormone stimulus was upregulated in PHY906 treated tumor tissues and we speculated that PHY906 induced cancer cell death by activating the response to steroid hormone stimulus.

The original paper suggested that PHY906 directly modulated the cancer cell survival in cell cycle and apoptosis in tumor cells, which is similar to our findings. PPI network analysis also showed that the genes in module 2 were closely related to cell cycle and genes in module 1 were significantly enriched in sensory perception of chemical stimulus. It is reported that PHY906 reduces chemotherapy-induced gastrointestinal toxicity in colon cancer treatment [4]. The cell cycle and sensory perception of chemical stimulus were altered in tumor tissues induced by PHY906, which further indicated that the PHY906 could mediate cancer cell death by modulating cell cycle and sensory perception of chemical stimulus.

Moreover, Ezh2 was found to be the key node in PPI network according to both degree and betweenness. Ezh2 was also clustered in module 2. A line of evidence showed that Ezh2 played key role in stimulating cell growth and proliferation in colon cancer [24]. The expression of Ezh2 is reported to be closely related to treatment response and prognosis of colon cancer patients [25]. Ezh2 has been proposed to be therapeutic target for colon cancer intervention [24]. Besides, Ezh2 is predicted to be regulated by the 3 TFs in module 2, such as E2f1, Hsfy2, and Nfyb. E2f1 is a proliferation promoting TF and has been found to be accumulated in colon cancer [26]. HSF family members have been found to play key roles in proteostasis of cancers [27], while the role of Nfyb in colon cancer has been reported rarely. Thus, the TFs of E2f1, Hsfy2, and Nfyb may play key roles in colon cancer and the functions should be further studied.

Despite the significant findings in this paper, the microarray data analyzed were derived from murine colon tumor tissues. It is unknown whether the similar findings can be produced over human colon cancer tissues. It is a limitation in the present study and further analyses based on human samples are warranted in the near future.

In conclusion, PHY906 showed characteristic in controlling cell apoptosis by intervening interferon-gamma production and response to steroid hormone stimulus. The significant node of Ezh2 and its TFs such as E2f1, Hsfy2, and Nfyb may show the potential therapeutic effect on colon cancer. Our study may provide insights to understand the role of PHY906 in treating colon cancer and provide valuable information for novel anticancer agents development in the future. However, further studies are urgently needed.

## Figures and Tables

**Table 1:** The overrepresented GO terms and pathways for differentially expressed genes.

	Category	Term	Count	*P* value
Up	BP	GO:0048545~response to steroid hormone stimulus	6	4.38*E* − 03
		GO:0007131~reciprocal meiotic recombination	3	1.06*E* − 02
		GO:0000279~M phase	11	3.21*E* − 02
	CC	GO:0015629~actin cytoskeleton	10	1.01*E* − 02
		GO:0005578~proteinaceous extracellular matrix	12	1.56*E* − 02
		GO:0044449~contractile fiber part	6	1.74*E* − 02
	MF	GO:0015082~di- and trivalent inorganic cation transmembrane transporter activity	5	2.22*E* − 03
		GO:0017076~purine nucleotide binding	46	1.47*E* − 02
		GO:0030674~protein binding, bridging	4	1.95*E* − 02

Down	BP	GO:0032649~regulation of interferon-gamma production	4	6.74*E* − 03
		GO:0019439~aromatic compound catabolic process	3	1.39*E* − 02
		GO:0048535~lymph node development	3	2.19*E* − 02
	CC	GO:0000267~cell fraction	18	1.62*E* − 03
		GO:0005624~membrane fraction	16	2.28*E* − 03
		GO:0005626~insoluble fraction	16	3.17*E* − 03
	MF	GO:0005416~cation:amino acid symporter activity	3	6.38*E* − 03
		GO:0005506~iron ion binding	11	1.02*E* − 02
		GO:0015171~amino acid transmembrane transporter activity	4	2.37*E* − 02
	Pathway	mmu00380:tryptophan metabolism	5	3.10*E* − 03

Up means upregulated genes, and down represents downregulated genes.

**Table 2:** Top 10 significant nodes based on three network centralities.

Nodes	Subgraph	Nodes	Degree	Nodes	Betweenness
Olfr20	9011718	Acly	36	Acly	28651.502
Olfr1022	9011523	Rhoa	25	Rhoa	22965.008
Olfr281	8964057	Erbb3	25	Rps27a	13976.256
Olfr26	8964057	Rad51	23	Trip13	12352.914
Olfr1100	8964057	Ezh2	22	Tex11	11707.876
Olfr1463	8964057	Ccnb2	21	Erbb3	10906.968
Olfr1054	8964057	Rps27a	21	Olfr20	10419.8125
Olfr1032	8964057	Olfr20	20	Anln	8507.561
Olfr16	8964057	Olfr1022	20	Mmp2	8288.151
Olfr301	8964057	Rrm1	20	Ezh2	7548.0337

**Table 3:** The significant GO terms and pathways for genes in modules.

	Category	Term	Count	*P* value
Module 1	BP	GO:0007608~sensory perception of smell	20	2.10*E* − 21
		GO:0007606~sensory perception of chemical stimulus	20	7.28*E* − 21
		GO:0007600~sensory perception	20	1.62*E* − 19
	CC	GO:0016021~integral to membrane	20	3.34*E* − 07
		GO:0031224~intrinsic to membrane	20	6.53*E* − 07
	MF	GO:0004984~olfactory receptor activity	20	4.36*E* − 21
	Pathway	mmu04740:olfactory transduction	16	2.06*E* − 12

Module 2	BP	GO:0007049~cell cycle	10	4.37*E* − 08
		GO:0006259~DNA metabolic process	9	4.45*E* − 08
		GO:0006260~DNA replication	6	1.68*E* − 06
	CC	GO:0005694~chromosome	6	3.93*E* − 05
		GO:0044427~chromosomal part	5	3.36*E* − 04
		GO:0043228~non-membrane-bounded organelle	8	0.002514
	MF	GO:0003677~DNA binding	9	7.05*E* − 05
		GO:0005524~ATP binding	7	0.001417
		GO:0032559~adenyl ribonucleotide binding	7	0.001508
	Pathway	mmu03030:DNA replication	3	0.001267
		mmu03420:nucleotide excision repair	3	0.00191
		mmu04110:cell cycle	3	0.016042

## References

[1] Ferlay J., Soerjomataram I., Dikshit R. (2015). Cancer incidence and mortality worldwide: sources, methods and major patterns in GLOBOCAN 2012. *International Journal of Cancer*.

[2] Ricci-Vitiani L., Lombardi D. G., Pilozzi E. (2007). Identification and expansion of human colon-cancer-initiating cells. *Nature*.

[3] Tan K. Y., Liu C. B., Chen A. H., Ding Y. J., Jin H. Y., Seow-Choen F. (2008). The role of traditional Chinese medicine in colorectal cancer treatment. *Techniques in Coloproctology*.

[4] Lam W., Bussom S., Guan F. (2010). The four-herb Chinese medicine PHY906 reduces chemotherapy-induced gastrointestinal toxicity. *Science Translational Medicine*.

[5] Ye M., Liu S.-H., Jiang Z., Lee Y., Tilton R., Cheng Y.-C. (2007). Liquid chromatography/mass spectrometry analysis of PHY906, a Chinese medicine formulation for cancer therapy. *Rapid Communications in Mass Spectrometry*.

[6] Farrell M. P., Kummar S. (2003). Phase I/IIA randomized study of PHY906, a novel herbal agent, as a modulator of chemotherapy in patients with advanced colorectal cancer. *Clinical Colorectal Cancer*.

[7] Wang E., Bussom S., Chen J. (2011). Interaction of a traditional Chinese Medicine (PHY906) and CPT-11 on the inflammatory process in the tumor microenvironment. *BMC Medical Genomics*.

[8] Smyth G. K. (2005). *Limma: Linear Models for Microarray Data. Bioinformatics and Computational Biology Solutions Using R and Bioconductor*.

[9] Huang D. W., Sherman B. T., Lempicki R. A. (2009). Systematic and integrative analysis of large gene lists using DAVID bioinformatics resources. *Nature Protocols*.

[10] Szklarczyk D., Franceschini A., Wyder S. (2015). STRING v10: protein-protein interaction networks, integrated over the tree of life. *Nucleic Acids Research*.

[11] Tang X., Wang J., Zhong J., Pan Y. (2014). Predicting essential proteins based on weighted degree centrality. *IEEE/ACM Transactions on Computational Biology and Bioinformatics*.

[12] Goh K. I., Oh E., Kahng B., Kim D. (2003). Betweenness centrality correlation in social networks. *Physical Review E*.

[13] Estrada E., Rodríguez-Velázquez J. A. (2005). Subgraph centrality in complex networks. *Physical Review E*.

[14] Tang Y., Li M., Wang J., Pan Y., Wu F.-X. (2015). CytoNCA: a cytoscape plugin for centrality analysis and evaluation of protein interaction networks. *BioSystems*.

[15] Nepusz T., Yu H., Paccanaro A. (2012). Detecting overlapping protein complexes in protein-protein interaction networks. *Nature Methods*.

[16] Janky R., Verfaillie A., Imrichová H. (2014). iRegulon: from a gene list to a gene regulatory network using large motif and track collections. *PLoS Computational Biology*.

[17] Qi F., Li A., Inagaki Y. (2010). Chinese herbal medicines as adjuvant treatment during chemo-or radio-therapy for cancer. *Bioscience Trends*.

[18] Madara J. L., Stafford J. (1989). Interferon-*γ* directly affects barrier function of cultured intestinal epithelial monolayers. *The Journal of Clinical Investigation*.

[19] Blum M. S., Toninelli E., Anderson J. M. (1997). Cytoskeletal rearrangement mediates human microvascular endothelial tight junction modulation by cytokines. *American Journal of Physiology—Heart and Circulatory Physiology*.

[20] Kiess W., Gallaher B. (1998). Hormonal control of programmed cell death/apoptosis. *European Journal of Endocrinology*.

[21] Michna H., Nishino Y., Neef G., McGuire W. L., Schneider M. R. (1992). Progesterone antagonists: tumor-inhibiting potential and mechanism of action. *Journal of Steroid Biochemistry and Molecular Biology*.

[22] Herr I., Debatin K.-M. (2001). Cellular stress response and apoptosis in cancer therapy. *Blood*.

[23] Milas L., Stephens L., Meyn R. (1993). Relation of apoptosis to cancer therapy. *In Vivo*.

[24] Fussbroich B., Wagener N., Macher-Goeppinger S. (2011). EZH2 depletion blocks the proliferation of colon cancer cells. *PLoS ONE*.

[25] Fluge O., Gravdal K., Carlsen E. (2009). Expression of EZH2 and Ki-67 in colorectal cancer and associations with treatment response and prognosis. *British Journal of Cancer*.

[26] Murillo G., Salti G. I., Kosmeder J. W., Pezzuto J. M., Mehta R. G. (2002). Deguelin inhibits the growth of colon cancer cells through the induction of apoptosis and cell cycle arrest. *European Journal of Cancer*.

[27] Dai C., Sampson S. B. (2016). HSF1: guardian of proteostasis in cancer. *Trends in Cell Biology*.

